# In Vitro Reconstitution of the Melanin Pathway’s Catalytic Activities Using Tyrosinase Nanoparticles

**DOI:** 10.3390/ijms24010639

**Published:** 2022-12-30

**Authors:** Isabella Osuna, Monika B. Dolinska, Yuri V. Sergeev

**Affiliations:** National Eye Institute, National Institutes of Health, Bethesda, MD 20891, USA

**Keywords:** melanogenic pathway, oculocutaneous albinism, intra-melanosomal domains of tyrosinases, tyrosinase domain immobilized to magnetic beads, native dopachrome

## Abstract

The melanogenesis pathway is characterized by a series of reactions catalyzed by key enzymes, such as tyrosinase (TYR), tyrosinase-related protein 2 (TYRP2), and tyrosinase-related protein 1 (TYRP1), to produce melanin pigment. However, in vitro studies of the catalytic activity were incomplete because of a lack of commercially available enzyme substrates, such as dopachrome. Herein, human recombinant intra-melanosomal domains of key enzymes were produced in *Trichoplusia ni (T. ni)* larvae and then purified using a combination of chromatography techniques in catalytically active form. Using Michaelis–Menten kinetics, the diphenol oxidase activity of tyrosinase achieved the maximum production of native dopachrome at 10 min of incubation at 37 °C for TYR immobilized to magnetic beads (TYR-MB). The presence of dopachrome was confirmed spectrophotometrically at 475 nm through HPLC analysis and in the TYRP2-catalyzed reaction, yielding 5,6-dihydroxyindole-2-carboxylic acid (DHICA). In the TYRP1-driven oxidation of DHICA, the formation of 5,6-indolequinone-2-carboxylic acid (IQCA) was confirmed at ~560 nm. This is the first in vitro reconstitution of the reactions from the melanogenic pathway based on intra-melanosomal domains. In the future, this approach could be used for quantitative in vitro analysis of the melanin pathway, biochemical effects associated with inherited disease-related mutations, and drug screens.

## 1. Introduction

Tyrosinase and tyrosinase-related proteins 2 and 1 are key enzymes responsible for regulating the melanogenesis pathway that produces melanin in cells of the human eye skin, and hair. When the pathway is disrupted by inherited mutations of any of the above proteins, it can lead to a lack or reduced production of melanin in the cell. Such a change could result in oculocutaneous albinism (OCA) which is linked to protein stability and enzyme activity [1]. Currently, there are seven different types of OCA caused by genetic alterations in different proteins: TYR (OCA1), OCA2-Melanosomal Transmembrane Protein (OCA2), TYRP1 (OCA3), Soluble Carrier Family 45 Member 2 (SLC45A2)/Membrane Associated Transporter, MATP (OCA4), Soluble Carrier Family 24 Member 5 (SLC24A5) (OCA6), and Leucine-Rich Melanocyte Differentiation Associated (LRMDA) (OCA7). Recently, dopachrome tautomerase (DCT/TYRP2) has been linked to eight subtypes of OCA, which is proposed to be named OCA8 [2]. To date, only one family has been reported with OCA5 disorder, linked to markers on chromosome 4q24, but a singular “OCA5 gene” and corresponding protein have yet to be established [3]. The subtypes of OCA are categorized with similar and overlapping phenotypes, such as ocular abnormalities, hypopigmentation, and decreased visual capabilities. Therefore, to clinically diagnose and differentiate between them, genetic testing is required, and further analysis of the melanogenesis pathway is crucial.

Cutaneous melanin pigment plays a critical role in protection against the harmful effects of solar radiation and other factors. Melanogenesis is under complex regulatory control by multiple agents interacting via pathways activated by receptor-dependent and -independent mechanisms, in hormonal, auto-, para-, or intracrine fashion [4]. The most important positive regulator of melanogenesis is the MC1 receptor with its ligands, melanocortins, and ACTH, whereas among the negative regulators, agouti protein stands out, determining the intensity of melanogenesis and also the type of melanin synthesized. According to the modern view, melanogenic activity performs the functions of a unique molecular sensor, the signal transducer, and could work as the regulator of local homeostasis. In keeping with these multiple roles, melanogenesis is controlled by a highly structured system, active since early embryogenesis, and capable of super-selective functional regulation that may reach down to the cellular level, represented by single melanocytes.

Additionally, in these processes, tyrosine and L-DOPA are performing functions of substrates and intermediates of enzymatic reactions. Furthermore, these molecules perform the roles of inducers and positive regulators of melanogenesis and in other cellular functions. The substrate-induced melanogenic pathway would autoregulate itself as well as regulate the melanocyte functions through the activity of its structural or regulatory proteins and through intermediates of melanogenesis and melanin itself [5].

**Figure 1:**
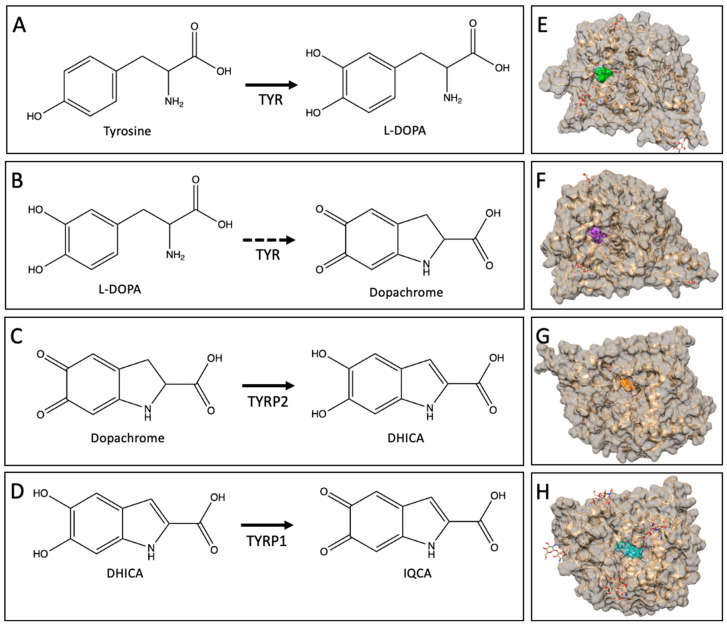
Key steps in melanogenesis pathway. (**A**) TYR catalyzes the conversion of tyrosine to L-DOPA. (**B**) TYR catalyzes L-DOPA to the intermediate dopaquinone (not pictured) to dopachrome. (**C**) TYRP2 catalyzes the conversion of dopachrome to DHICA. (**D**) TYRP1 catalyzes the conversion of DHICA to IQCA. The reactions are labeled as follows: TYR + L-DOPA, diphenol oxidation, and spontaneous conversion of dopaquinone to dopachrome; TYRP2 + dopachrome, dopachrome tautomerization; TYRP1+ DHICA, the oxidation of DHICA to IQCA. Panels E–H are visualizations done in CHIMERA (Version 1.15.0) with the surface of tyrosinases shown in beige. (**E**) Tyrosine, shown in green, docked to the active site. (**F**) L-DOPA, shown in purple, is docked in the active. (**G**) Dopachrome, in orange, is docked in TYRP2. (**H**) DHICA, shown in cyan, is docked to TYRP1.

In the eye, the melanogenesis pathway can be summarized by a series of four major reactions, shown in Figure 1, catalyzed by the three key tyrosinase-like enzymes. In the presence of molecular oxygen, TYR functions to convert amino acid tyrosine to levodopa (L-DOPA) in a monophenol hydroxylation reaction (not studied in this report), followed by catalyzing the diphenol oxidation of L-DOPA to dopaquinone at the beginning of the pathway. Dopaquinone, a chemically unstable molecule, undertakes intramolecular cyclization and oxidation to form a dopachrome [6]. At the second step of the pathway, TYRP2 catalyzes the tautomerization of pigmented intermediate dopachrome to DHICA. In the third reaction, TYRP1 catalyzes the oxidation of DHICA to IQCA. At the ending stages of the pathway, the reaction of 5,6-dihydroxyindole (DHI) and DHICA forms indole-5,6-quinone (IQ) and IQCA, respectively, which polymerize to yield eumelanin. Eumelanin is one of the two types of melanin, a brown–black insoluble polymer that is more efficient at photoprotection, while pheomelanin, a red–yellow soluble polymer, is more common in skin types susceptible to skin tumors [7,8].

Mutations in TYR are linked to two types of OCA1, OCA1A leading to no tyrosinase activity and OCA1B leading to decreased enzymatic activity. When mutants of both subtypes were purified and biochemically analyzed, mutations causing OCA1A showed little expression and no activity, while mutations causing OCA1B showed similar expression to wild-type and lower activity and stability [1]. Additional studies investigating the temperature dependency of the TYR reaction with its substrate, L-DOPA, showed that the diphenol oxidase activity is a spontaneous, enthalpy-driven reaction that loses favorability once the product, dopachrome, is formed [9]. Further investigation into temperature-dependent kinetics demonstrated that two mutants, R422Q and P406L, caused OCA1B to have a lower enzyme turnover rate compared to the wild-type, but production of dopachrome increases with increasing temperature for both mutants as well as TYR [10].

The product of the TYR reaction, native dopachrome, was obtained using human recombinant TYR intra-melanosomal domains immobilized to magnetic beads for further studies and characterization of the isolated product [11]. In this work, a method to isolate native dopachrome using magnetic beads was established and showed that the immobilized TYR is a stable and catalytically active protein and retains stability at a higher temperature when intact tyrosinase shows no catalytic activity. The protein remains active according to Hill kinetics and native dopachrome production increases with increasing temperature or the number of magnetic beads. Ordinarily, magnetic beads coated with nickel-charged nitrilotriacetic acid (Ni-NTA) are used in protein purification. However, based on this work, we utilized the affinity of the his-tagged protein with nickel to immobilize TYR to the beads and introduced the substrate L-DOPA to the immobilized TYR, which allowed us to isolate our product, native dopachrome, and model the TYR reaction in vitro.

In the reaction of tautomerization, TYRP2 catalyzes the production of DHICA from dopachrome. Unfortunately, the investigation into TYRP2 is limited because dopachrome is not commercially available. Therefore, we decided to use native dopachrome isolated from the TYR-MB reaction obtained in vitro for the analysis of the enzymatic activity of TYRP2. This is especially important for the comparison of catalytic activities of the TYRP2-associated mutants. Recently, C40S, C61W, and G59V were identified as causing OCA8 and were linked to either complete protein instability or decreased enzymatic activity [2,12,13]. Previously, we used native dopachrome and human recombinant intra-melanosomal domain of TYRP2 to demonstrate that the TYRP2 mutations (C40S and C61W) and different truncations of the protein caused the misfolding and instability that ultimately led to the loss of activity and function of the enzyme [12].

The role of TYRP1 in the melanogenesis pathway is to catalyze the oxidation of DHICA to IQCA. Previously, it has been reported that TYRP1 acts as a chaperone to TYR in the endoplasmic reticulum (ER), which increases TYR stability and suggests that the two proteins form a dimer within the melanosome [14,15]. However, our group reported the increased function and stability of the TYR intra-melanosomal domain when in the presence of excess TYRP1 in vitro at different temperatures and pH to mimic the environment of the ER and melanosomes without the formation of a stable multi-enzyme complex, which contradicted previous studies [16]. Further biochemical investigation into TYRP1, with conditions to replicate the ER and melanosome, showed the protein at a monomeric state at temperatures equal to or less than 25 °C but then showed that aggregates of TYRP1 form when temperatures reach and exceed 31 °C [17]. Additionally, the activity of TYRP1 to oxidize DHICA increased with temperature and was shown to be dependent upon the pH, the use of 3-methyl-2-benzothiazolinone hydrazone hydrochloride (MBTH), and the inclusion of a reducer [17]. Computational studies on TYRP1 and four mutants (C30R, H215Y, D308N, and R326H) revealed larger binding clefts present in C30R and H215Y mutants. The binding clefts may have resulted in the loss of stability that was predicted with the unfolding mutation screen [18,19]. The predictions were experimentally confirmed, compared to D308N and R326H mutants wherein hydrogen bonding and salt bridge interactions help to stabilize the substrate (DHICA) in the active site of TYRP1, D308N, and R326H [20].

In our group, we expressed and purified the intra-melanosomal domains of human recombinant tyrosinases, TYR, TYRP1, and TYRP2 [12,16,21]. The intra-melanosomal domains were obtained by the removal of the C-terminal parts of TYR, TYRP1, and TYRP2 involving the trans-membrane helices. All recombinant domains of tyrosinases demonstrate their catalytic activities in vitro, which have been reported in the literature and presented in Figure 1. In the future, one compelling idea is to reconstitute the entire melanosomal pathway in vitro using the intra-melanosomal domains of tyrosinases. However, analysis of the pathway is hindered because not all substrates in the melanogenesis pathway are stable chemical molecules and/or commercially available. Using the enzymes from the pathway immobilized to magnetic beads might help to analyze the different steps of the melanogenic pathway using native substrates, such as dopachrome, DHICA, and others, prepared in vitro in ‘native’ conditions of recombinant protein domains.

Herein, we expand on our previous study and characterize the enzymatic activity of TYR immobilized to magnetic beads at various time points, to optimize the production of the native dopachrome in vitro and demonstrate that the maximum amount of dopachrome is produced at 10 min of incubation. The dopachrome produced in the tyrosinase reaction was converted to DHICA through the dopachrome tautomerase reaction by TYRP2, which was demonstrated spectrophotometrically and compared to the commercial form of DHICA. Additionally, the oxidation of DHICA into IQCA by TYRP1 has previously only been demonstrated with the use of MBTH. However, we were able to confirm the activity of TYRP1 with the formation of IQCA without the use of MBTH for the first time. In summary, we demonstrated the success of dopachrome as a substrate through the characterization of TYR-MB particles activity, confirmed the enzymatic activity of both TYRP2 and TYRP1, and identified the substrates in the melanogenesis pathway through HPLC analysis.

## 2. Results

### 2.1. Protein Purificaiton

**Figure 2:**
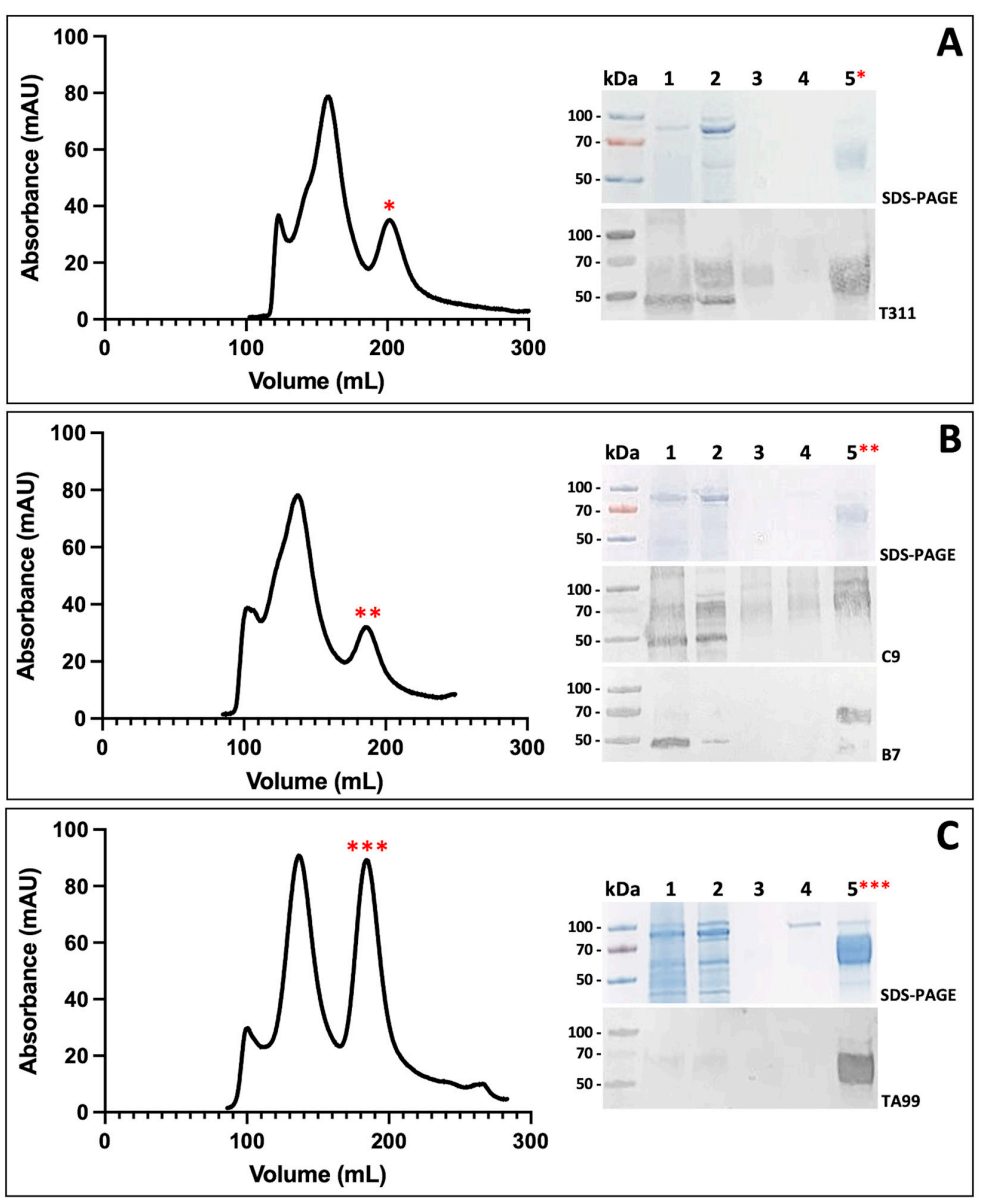
Purification profiles of lysates of recombinant human tyrosinases after 2nd step of purification using ÄKTAxpress. SEC profiles of TYR, TYRP2, and TYRP1 are shown in Panels (**A**–**C**), respectively. The inserts show the SDS-PAGE (top) and Western blot (bottom) analyses of the pellet before purification (1), lysate before purification (2), and the three peaks (3, 4, and 5). A protein of interest was identified in the third peak from the left (red asterisks) with an expected weight of ~60 kDa. T311, anti-TYR antibody; C9 and B7, anti-TYRP2 antibodies; TA99, anti-TYRP1 antibody.

Recombinant tyrosinases (TYR, TYRP2, and TYRP1) were purified using an ÄKTAxpress chromatography system and utilized immobilized metal affinity chromatography (IMAC) and size-exclusion chromatography (SEC) as demonstrated previously [12,16,21,22]. In the SEC profile, the samples were eluted into three different peaks (Figure 2). The lysate, pellet, and peaks were then analyzed for the desired protein using SDS-PAGE and Western blot analyses. TYR was identified in the third chromatography peak (Figure 2A) at the expected band length of ~59 kDa (Figure 2A Insert). The TYR fractions (190–230 mL) were pooled and concentrated (5.96 mg). TYRP2 was identified in the third peak (Figure 2B) at the expected band length of ~58 kDa (Figure 2B Insert). The TYRP2 fractions (175–208 mL) were pooled and concentrated (4.03 mg). TYRP1 was identified in the third peak (Figure 2C) at the expected band length of ~59 kDa (Figure 2C Insert). The TYRP1 fractions (168–235 mL) were pooled and concentrated (15.41 mg). The activity of TYR, TYRP2, and TYRP1 was confirmed as described below.

### 2.2. Michaelis Menten Kinetics of TYR-MB Nanoparticles

**Figure 3:**
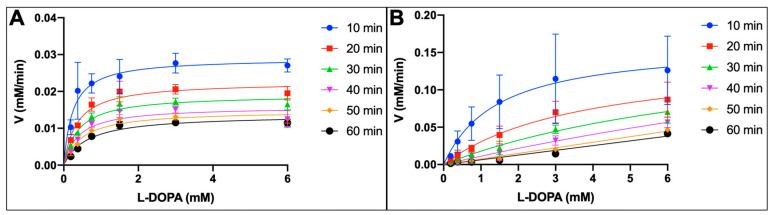
The maximum velocity (V_max_) of diphenol oxidase reaction as a function of L-DOPA concentration for control and TYR-MB nanoparticles. (**A**) The control TYR. (**B**) TYR-MB nanoparticles. The measurement was repeated from 10 to 60 min at 10 min intervals. For each time point, the measurements of the V_max_ were obtained in triplicates and averaged, and standard errors were calculated.

Tyrosinase nanoparticles were prepared as described in the Methods Section 4.2. Michaelis–Menten kinetics were measured for TYR-MB nanoparticles and intact TYR (control). For both samples, the maximum velocity of the diphenol oxidase reaction was recorded every 10 min for 60 min as a function of L-DOPA concentration and plotted (Figure 3). The activity of control TYR (Figure 3A) was measured using Beer’s law (A = εcl) at an absorbance of 475 nm and fitted using the Michaelis–Menten equation Y = (V_max_*x)/(K_m_ + x). Additionally, the activity of TYR-MB nanoparticles (Figure 3B) was also fitted using Michaelis–Menten for the first time. As the concentration of substrate (L-DOPA) increases, both reactions reach a saturation point (maximum velocity, V_max_).

The reaction parameters of the diphenol oxidase reaction were calculated from the plots in Figure 3 and compared for the control TYR and TYR-MB reactions at 30 min of incubation with the standard error (Table 1). The V_max_ of the TYR + L-DOPA reaction increases when TYR is immobilized. The Michaelis constant (K_m_) increases, which shows that the affinity decreases when tyrosinase nanoparticles are used in the reaction. The enzyme turnover rate (k_cat_) increases 5-fold when TYR is bound to the magnetic beads. However, the enzyme efficiency (k_cat_/K_m_) decreases when TYR is immobilized compared to the control.

### 2.3. Optimum Conditions for Native Dopachrome Production

**Figure 4:**
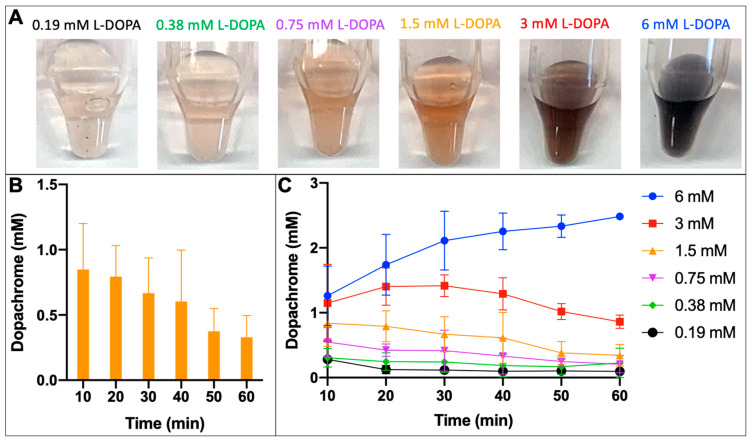
Isolated native dopachrome products of TYR-MB reaction. (**A**) The increasing concentrations of L-DOPA (left to right) show a change in color from clear through orange to black at 10 min of incubation. (**B**) When using 1.5 mM L-DOPA, the production of dopachrome peaks at 10 min and then declines. (**C**) The concentration of dopachrome increases as the concentration of L-DOPA increases from 0.19–6 mM.

We monitored the dopachrome yield at each concentration of L-DOPA used every 10 min for 60 min to find the optimum conditions to produce native dopachrome (Figure 4). The different concentrations of L-DOPA produced a significant color change at the end of the reaction from clear through orange to black (Figure 4A). The color of dopachrome is a bright orange, achieved by using 1.5 mM L-DOPA. The higher concentrations (3 and 6 mM) of L-DOPA resulted in a black color, which indicates melanin-like products at the end of the melanogenesis pathway. The lack of color at the lower concentrations (0.19 and 0.38 mM) of L-DOPA indicated little to no dopachrome production. When 1.5 mM L-DOPA was used, the production of native dopachrome was at its maximum at 10 min (Figure 4B) of incubation and decreased after that as shown by the orange line (Figure 4B,C).

### 2.4. TYRP2 Enzymatic Activity

**Figure 5:**
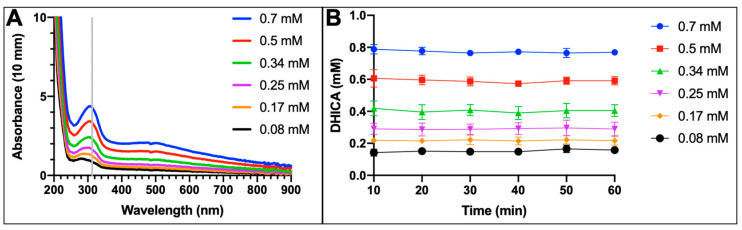
TYRP2 is enzymatically active. (**A**) In the dopachrome tautomerase reaction, TYRP2 incubated with isolated native dopachrome for 30 min causes an increase in the DHICA absorbance at 315 nm (noted with the gray bar). Increasing concentrations of dopachrome from 0.08–0.7 mM were used. (**B**) The concentration of DHICA increases with increasing concentrations of substrate used but remains constant over the 1 h reaction.

The dopachrome tautomerase reaction is the third step of the melanogenic pathway (Figure 1C). In this reaction, to determine the enzymatic activity of TYRP2, the protein was incubated with the native dopachrome isolated with the TYR-MB protocol. The activity of TYRP2 was observed with an increase in absorbance at 315 nm, the maximum absorption for the DHICA molecule when concentrations of dopachrome were increased stepwise in the reaction (Figure 5A).

The activity of the product of the TYRP2 reaction was confirmed using the commercial DHICA reagent and demonstrated a peak of absorbance at the same wavelength of 315 nm (Supplementary Figure S1B). This concludes that the absorbance of the product formed in the TYRP2 and native dopachrome reaction matches the absorbance of the commercial product of DHICA (Figure 5A and Figure S1B), suggesting that native dopachrome can be used as a substrate in the TYRP2 reaction. This conclusion was further confirmed in the experiment when the concentration of native dopachrome was increased, which caused a larger peak at 315 nm, which indicates a higher concentration of DHICA forming (Figure 5). Consequently, the concentration of DHICA at a wavelength of 315 nm was calculated for each concentration throughout the 1 h reaction (Figure 5B). In our attempts at Michaelis–Menten analysis, the velocity of the reaction did not reach a maximum point, indicating that the active site of the enzyme was not fully saturated despite using the highest concentration of dopachrome achieved in the TYR-MB isolation.

### 2.5. TYRP1 Enzymatic Activity

**Figure 6:**
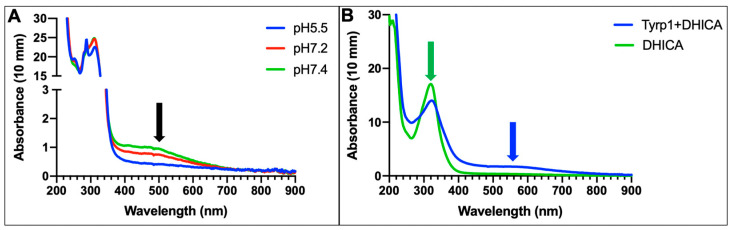
TYRP1 is Enzymatically Active. (**A**) TYRP1 was incubated for 4 hr with DHICA (1.5 mM), MBTH (1.5 mM), and TCEP (25 µM) at a pH of 5.5 (blue line), 7.2 (red line), and 7.4 (green line), which produces a peak at ~505 nm (black arrow) at 37 °C. (**B**) TYRP1 reacted with DHICA (blue line) and produces a broad peak representing the formation of a new product (IQCA) at ~560 nm (blue arrow) after 24 h incubation at 37 °C. The substrate decreases in absorbance at ~325 nm (green arrow) after 24 h incubation with TYRP1.

The activity of TYRP1 was initially confirmed using MBTH, which forms a dye complex with IQCA, and this new product forms a peak at 505 nm (Figure 6A) [23]. In our case, we performed this reaction in the presence of a reducer tris(2-carboxyethyl)phosphine hydrochloride (TCEP). This reaction was tested for 4 h at a pH of 5.5, 7.2, and 7.4. However, the TYRP1 activity was also tested in the absence of MBTH and confirmed with the addition of the substrate, DHICA at 1.5 mM, for 24 h at 37 °C and shaking at 270 RPM. After the incubation, the spectrum of the reaction shows the formation of a peak corresponding to a new product (IQCA) at ~560 nm (Figure 6B). This reaction was performed using increasing concentrations of DHICA (not shown) to calculate the enzymatic characteristics using Michaelis–Menten. Nevertheless, the reaction did not reach a maximum velocity despite using the highest concentration of DHICA, therefore Michaelis–Menten was unable to be utilized. The concentration was limited to 3 mM due to the poor solubility of DHICA in water.

### 2.6. Substrates Identified through HPLC

**Figure 7:**
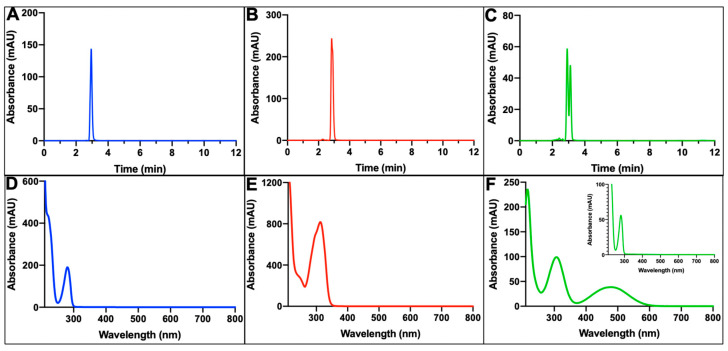
L-DOPA, DHICA, and native dopachrome are identified through HPLC. (**A**) The standard of L-DOPA (1.5 mM) has a retention time of 2.939 ±  0.024 min. (**B**) The standard of DHICA (1.5 mM) has a retention time of 2.864 ±  0.054 min. (**C**) The sample of native dopachrome was eluted with two retention times at 2.913 ±  0.024 min and 3.110 ±  0.027 min. (**D**) The peak at 2.939 retention time has an absorbance spectrum with a peak at 280 nm. (**E**) The peak at 2.864 retention time has an absorbance spectrum with a peak at 315 nm. (**F**) The peak at 2.913 retention time has an absorbance spectrum with a peak at 280 nm (seen in the insert). The peak at 3.110 retention time has an absorbance spectrum with peaks at 310 and 475 nm.

The three substrates, commercial L-DOPA, commercial DHICA, and native dopachrome, were independently identified through HPLC, and the retention times were averaged over three runs (Figure 7A–C). The two standards (L-DOPA and DHICA) were separated into one peak, while the native dopachrome prepared using TYR-MB was eluted from the column into two peaks (Figure 7C). The first compound to elute in our native sample was at a retention time of 2.913 min and is suspected to be L-DOPA. The second compound to elute was at a retention time of 3.110 min and is suspected to be dopachrome. The spectra of each peak match the previously reported spectra of each compound (Figure 7D–F and Figure S1).

## 3. Discussion

Human recombinant intra-melanosomal domains of TYR, TYRP1, and TYRP2 were produced in *T. ni* larvae and then purified using a combination of chromatography techniques in catalytically active form. We demonstrated that despite the immobilization of TYR, the protein remains enzymatically active while bound to the beads. We showed that the enzymatic activity of the TYR-MB reaction every 10 min for 1 h using Michaelis–Menten kinetics and that the maximum production of this reaction is achieved after 10 min of incubation at 37 °C.

The kinetic values calculated from Michaelis–Menten, shown in Table 1, showed an increasing V_max_ for the TYR-MB, which is evidence that the tyrosinase nanoparticle reaction requires more time to reach saturation. The affinity for the TYR-MB reaction decreases, evident with an increased K_m_. However, the enzyme turnover rate has a 5-fold increase in k_cat_, which shows the immobilized protein produces more product, which allows us to isolate our native dopachrome. Finally, the efficiency of the enzyme (k_cat_/K_m_) decreases with TYR-MB particles. We believe that the differences seen for the kinetic values in the TYR and TYR-MB reactions are due to the availability of the active site. When TYR is immobilized, the protein is restricted, and the substrate has limited availability to bind into the active site. To visualize this effect, we aim to simulate immobilization through molecular modeling in future studies.

After finding the maximum production of native dopachrome, through the activity of the TYR-MB reaction, we used this product as a substrate to test the activity of TYRP2, the next step in the melanogenesis pathway. TYRP2 catalyzed the conversion of our native dopachrome to DHICA, which was compared to the commercial form spectrophotometrically with a peak at 315 nm for both. Earlier studies have investigated the wavelength of DHICA and reported a peak shift from 313–318 nm when under different conditions [24].

For the first time, the TYR-MB reaction to isolate native dopachrome has been enzymatically and quantitatively characterized under different conditions to increase the efficiency of the reaction. Before using native dopachrome in the next enzymatic step, the purity was analyzed using HPLC, which showed two peaks representing L-DOPA and dopachrome in the sample (Figure 7C). This analysis showed that within our native sample, remnants of the substrate remained, but the suspected product was evident and used in the TYRP2 reaction. As expected, the increasing concentration of dopachrome we achieved allowed us to demonstrate that the TYRP2 activity is dependent on the concentration of substrate used. Despite increasing the concentration, Michaelis–Menten analysis was unable to be achieved because the TYRP2 activity did not reach its maximum velocity with the available concentration. Additionally, the limited concentration of dopachrome available will impact future studies investigating the ability to isolate native DHICA by immobilizing TYRP2 to magnetic beads, because, without increasing the amount of substrate available, isolating the product through immobilization will also be limited. Therefore, additional studies to further increase the concentration of dopachrome are underway.

To test the enzymatic activity of the third key enzyme in the melanogenesis pathway, the commercial DHICA was used as a substrate. The product of the TYRP1 oxidase activity has only previously been shown with the use of MBTH, which traps the product and forms a dye complex visible at 505 nm [25]. Other studies have investigated the role and activity of TYRP1 in an environment mimicking the pH of the ER and melanosome, which showed higher activity under melanosome conditions (pH 5.5), which contradicts this report [16,17]. However, earlier reports have not shown the activity of TYRP1 activity with only the substrate and enzyme in the reaction. In the present work, for the first time, we have shown that after 24 h of incubation at 37 °C, it is possible to observe the product of the TYRP1-catalyzed reaction (IQCA) identified spectrophotometrically with a broad peak with the maximum at 560 nm (Figure 6B). This proposes that the last step of the melanogenesis pathway might be a slow reaction.

Metal chelates in affinity chromatography have been used in protein biochemistry for a long time. At present, immobilized metal-affinity chromatography (IMAC) is a separation technique used for the isolation and purification of proteins. IMAC is based on the specific coordinate covalent bond of amino acids from a tag, such as a histidine tag, to metals immobilized in the chromatography resin. Further improvement in the affinity purification methods is possible after the introduction of mono-sized magnetic beads, which could use immobilized metal affinity for the selective binding of protein molecules. Such magnetic beads are widely available commercially from different vendors. We created magnetic beads designed for IMAC to generate immobilized metal-affinity nanoparticles containing an active human tyrosinase intra-melanosomal domain [11]. In the present work, the TYR-MB nanoparticles were prepared using a similar protocol and were used for the large-scale production of native dopachrome. The identity of dopachrome was confirmed spectrophotometrically at 475 nm (Figure 4A,B), through HPLC analysis (Figure 7C) and, in the TYRP2-catalyzed reaction, yielding DHICA (Figure 5A).

Although the preliminary study of dopachrome was performed previously in our work [11], we immediately freeze our dopachrome samples after isolation using a bath consisting of EtOH and dry ice. These samples were placed at −80 °C and have been used for up to 3 months after isolation. The native dopachrome samples, without the ice bath, have a shelf life of ~30 min because the oxidation will continue, and the samples will become black. We have confirmed that the −80 °C condition prevented autooxidation of dopachrome most effectively with the least melanin production compared to room temperature conditions and retained its orange–brown color for further use.

In summary, melanogenesis is a key step in the regulation of cellular metabolism. The important role of melanin and active melanogenesis was previously discussed [26,27]. In our work, we demonstrate the melanin pathway at each step and the individual reactions with the enzyme under the most native conditions to date, to mimic and replicate the in vivo conditions. Through our work utilizing native dopachrome as a substrate and confirming the activity by only including the enzyme and substrate in the reactions, we will be able to compare the reactions and activity when all three proteins are used in a single reaction. To further understanding of the melanogenesis pathway and enhance studies on treatments targeting this pathway for diseases such as OCA, we purified human recombinant tyrosinases involved in regulating the melanin pathway, isolated key substrates, and characterized the diphenol oxidase activity of tyrosinase and confirmed the activity of TYRP1 and TYRP2 to develop an in vitro model. By continuing our investigation into this reaction, we hope to apply this isolation method to each step in the melanogenesis pathway to produce a native form of each substrate to apply in the reaction. The native form of substrates will allow us to better mimic the pathway by avoiding commercial products and relying solely on the enzymes’ role in melanogenesis. We hope that this is the first in vitro reconstitution of the melanogenic pathway based on catalytically active recombinant protein domains. In the future, this reconstitution system would be useful for quantitative in vitro analysis of the melanin pathway, biochemical effects associated with inherited disease-related mutations, and drug design.

## 4. Materials and Methods

### 4.1. Protein Purification

Recombinant truncated tyrosinases (TYR, TYRP2, and TYRP1) were expressed in baculovirus and produced in *T. ni* larvae (Allotropic Tech, LLC, Halethorpe, MD, USA). First, larval biomass for each protein was homogenized in 5x lysate buffer (20 mM sodium phosphate, pH 7.4, 500 mM NaCl, 5 mM imidazole, 25 μM 1-phenyl-2-thiourea, PTU (Sigma-Aldrich, Saint Louis, MO, USA), 2 mM MgCl_2_, 40 μg/mL DNAse I (Thermo Fischer Scientific, Waltham, MA, USA), 0.2 mg/mL lysozyme, and protease inhibitors (Roche, San Francisco, CA, USA). The lysate was then incubated at 25 °C for 30 min, sonicated at 25 °C for 10 min, and centrifuged at 4 °C for 30 min at 8000 RPM before dilution with affinity binding buffer (20 mM sodium phosphate, pH 7.4, 500 mM NaCl, 20 mM imidazole).

The proteins were purified by IMAC using a His-Trap 5 mL Crude Column (GE Healthcare, NJ, USA) and SEC using a HiPrep 26/60 Sephacryl S-300 column. The columns were run on a ÄKTAxpress liquid chromatography system and analyzed using UNICORN software (GE Healthcare, Silver Spring, MD, USA). The samples were loaded onto the IMAC column with affinity binding buffer, eluted with affinity elution buffer (20 mM sodium phosphate, pH 7.4, 500 mM NaCl, 500 mM imidazole), and then applied onto the SEC column with gel filtration buffer (50 mM Tris-HCl, pH 7.4, 1 mM ethylenediaminetetraacetic acid (EDTA), 50 μM TCEP, 150 mM NaCl). All three lysates eluted into three peaks and TYR, TYRP2, and TYRP1 identities were confirmed by SDS-PAGE and Western blot analyses using anti-TYR antibodies (T311, Santa Cruz Biotechnology, Dallas, TX, USA), anti-TYRP2 (C9 and B7, Santa Cruz Biotechnology, Dallas, TX, USA) antibodies, and anti-TYRP1 antibodies (TA99, Santa Cruz Biotechnology, Dallas, TX, USA). The identified TYR fractions (190–230 mL) were collected, pooled, and concentrated with a final amount of ~6 mg and stored at −80 °C until further use. The identified TYRP2 fractions (175–208 mL) were also pooled and concentrated with a final amount of ~4 mg and stored at −80 °C until further use. The identified TYRP1 fractions (168–235 mL) were pooled and concentrated to a final amount of ~15 mg and stored at −80 °C until further use.

### 4.2. Tyrosinase Nanoparticles and Enzymatic Activity

TYR at a final concentration of 0.25 mg/mL and L-DOPA (Sigma-Aldrich, Saint Louis, MO, USA) were reacted to measure the diphenol oxidase activity. Different concentrations of L-DOPA (6, 3, 1.50, 0.75, 0.38, and 0.19 mM) in 10 mM NaPO_4_ at pH 7.4 was incubated with TYR at 37 °C and shaken at 270 RPM for 60 min.

TYR (1 mg/mL) was immobilized to Ni-NTA (His-Tag Affinity) magnetic beads (Advanced Biochemicals, Lawrenceville, GA, USA) as previously described [11] and detailed below. TYR-MB was reacted with L-DOPA to isolate dopachrome. To find an optimal condition for dopachrome production, different concentrations of L-DOPA (6, 3, 1.50, 0.75, 0.38, and 0.19 mM) in 10 mM NaPO_4_ at pH 7.4 were added to separate the reactions and placed in an incubator at 37 °C and shaken at 270 RPM for 60 min. The spectrum of absorption from 200–900 nm was measured for both, control TYR and TYR-MB, every 10 min using the NanoPhotometer N60-Touch (Implen, Westlake Village, CA, USA). The peak absorbance of dopachrome, the product of the TYR reaction, was measured at 475 nm.

### 4.3. Michaelis-Menten Kinetics of TYR

The TYR diphenol oxidase activity was modeled using Michaelis–Menten kinetics as described before [12,22]. The diphenol oxidase reaction, performed in triplicates, was tested with different concentrations of L-DOPA (0.19, 0.38, 0.75, 1.50, 3, and 6 mM) and measured every 10 min for 60 min on the NanoPhotometer N60-Touch. The concentration of dopachrome was determined using the Beer–Lambert law (A = εcl) with A = 475 nm, ε = 3700 M^−1^cm^−1^, and l = 10 mm. The kinetic parameters (V_max_ and K_m_) were determined using GraphPad Prism software, version 8.1.2. The turnover number (k_cat_) was determined as the number of substrate (L-DOPA) molecules that transform into the product (dopachrome) per minute by the enzyme (TYR). The concentration of TYR was calculated using the sequence of amino acids by Expasy (https:/www.expasy.org/, accessed on 21 October 2022), with 10% glycosylation considered in the final molecular weight. TYR was determined to be 58.4 kDa.

### 4.4. Native Dopachrome Isolation

Ni-NTA coated magnetic beads were prepped for a binding incubation with two steps of washing and resuspension with binding buffer (1 mM imidazole, 0.5 M NaCl, 20 mM Tris-HCl, pH 8.0). TYR (1 mg/mL) was added, while the binding incubation was performed on a Roto-Mini Rotator (Benchmark Scientific, Sayreville, NJ, USA) at room temperature for 30 min. From there, the supernatant from the beads was collected using a magnetic separator (Qiagen, Germantown, MD, USA), and the concentration was measured to confirm a 10-fold decrease in concentration to ensure the protein was immobilized to the beads. The beads were washed twice with wash buffer (5 mM imidazole, 0.25 M NaCl, 0.05% Tween-20, 10 mM Tris-HCl, pH 8.0) and once with PBS before resuspension in PBS. The substrate at 1.5 mM L-DOPA in 10 mM NaPO_4_ at pH 7.4 was added and placed in an incubator at 37 °C and shaken at 270 RPM for 10 min. The absorbance spectrum at 200–900 nm was measured using the N60-Touch and the concentration was calculated using Beer’s law as previously described above in Section 4.3. The isolated sample of the product of the tyrosinase reaction was then immediately placed in an EtOH ice bath and stored at −80 °C until further use in the Tyrp2-catalyzed reaction.

### 4.5. TYRP2 Enzymatic Activity

Using the isolation technique described above, native dopachrome (0.08, 0.17, 0.25, 0.35, 0.50, and 0.70 mM) in 10 mM NaPO_4_ at pH 7.4 was used as a substrate with TYRP2 (0.5 mg/mL). Once isolated, native dopachrome was stored at −80 °C before the reaction. The viability of dopachrome as a substrate was confirmed pre- and post-ice bath before the reaction with TYRP2 by confirming the absorbance at 475 nm on the N60-Touch. The reaction of TYRP2 and native dopachrome was incubated at 37 °C and shaken at 270 RPM, and the spectrum of absorption from 200–900 nm was measured every 10 min for 60 min on the NanoPhotometer N60-Touch. The absorption of the dopachrome tautomerase reaction product (DHICA) was measured, and the concentration was estimated using Beer’s law with A = 315 nm, ε = 5530 M^−1^cm^−1^, and l = 10 mm. This reaction was performed independently in triplicates, and measurements were averaged.

### 4.6. TYRP1 Enzymatic Activity

TYRP1 (1.0 mg/mL) was reacted with 1.5 mM DHICA (Toronto Research Chemicals, Toronto, ON, Canada) that was diluted in buffer (50 mM Tris-HCl, 1 mM EDTA, 150 mM NaCl) at pH 5.5, 7.2, and 7.4. The different conditions of buffer for the experiments included the addition of 25 μM TCEP and/or 1.5 mM MBTH at each pH. This reaction was measured every hour for 4 h at 37 °C, shaking at 270 RPM. Additionally, TYRP1 (1.0 mg/mL) was only reacted with 1.5 mM DHICA that was diluted in buffer at pH 7.4 for 24 h at 37 °C shaking at 270 RPM. The spectrum from 200–900 nm was checked after the incubation on the NanoPhotometer N60-Touch, and the reaction was completed in triplicates, and measurements were averaged. The product of the reaction involving MBTH was measured at the peak of absorbance at 505 nm. The product of the ‘native’ reaction involving only TYRP1 and DHICA, IQCA, was measured at the absorbance peak at 560 nm.

### 4.7. High-Performance Liquid Chromatography (HPLC)

The substrates and products in the melanin pathway were identified using RP-HPLC on an Agilent 1260 Infinity II LC (Agilent, Santa Clara, CA, USA). A 2 µL sample was injected at a 1.0 mL/min flow rate on a Poroshell 120 Phenyl-Hexyl, 4.6 × 100 mm, 4 µm column (Agilent, Santa Clara, CA, USA) at 30 °C with a wavelength set for detection at 280 nm. The chromatograms were analyzed using the OpenLAB CDS 2.3.53 (Agilent, Santa Clara, CA, USA). The standard of L-DOPA was prepared in 10 mM of NaPO_4_ (pH 7.4) with a final concentration of 1.5 mM. The standard of DHICA was prepared in water with a final concentration of 1.5 mM. The native dopachrome was isolated from the magnetic beads with a 10 min reaction time and stored at −80 °C until each run. The samples were run with an isocratic mobile phase of 90% 10 mM NaPO_4_ (pH 6.0) and 10% methanol. The samples were run in triplicates, and the retention times were averaged.

## 5. Conclusions

In this report, for the first time, we provide an enzymatic overview of the intra-melanosomal domains of tyrosinases, TYR, TYRP2, and TYRP1 in the melanin pathway. Previously, studies on melanin biosynthesis have been limited due to the inability to obtain substrates commercially or to confirm the identity of the products. Herein, we showed the enzymatic activity of the TYR-MB reaction using Michaelis–Menten kinetics and the maximum production of this reaction was achieved after 10 min of incubation at 37 °C. Consequently, we demonstrated that, despite the immobilization of TYR, the protein remains enzymatically active while bound to the beads. After optimizing the production of native dopachrome, through the activity of the TYR-MB reaction, we used this product as a substrate to test the activity of TYRP2, the next key step in the melanogenesis pathway. Additionally, we demonstrated that the last step of the melanogenesis pathway is a slow reaction requiring a 24 h incubation of the reaction mixture of TYRP1 and DHICA. The system of the three domains of tyrosinases are mimicking the catalytic activities of tyrosinases involved in catalyzing the reactions in the melanin pathway. Results of our work suggest in the future a simple catalytically active system for quantitative in vitro analysis of the melanin pathway, biochemical effects associated with inherited disease-related mutations, and the possibility of drug screening.

## Figures and Tables

**Table 1:** Michaelis–Menten kinetic parameters of diphenol oxidase reaction.

	V_max_ (mM/min)		K_m_ (mM^−1^)		k_cat_ (min^−1^)		k_cat_/K_m_ (min^−1^/mM)	
Time (min)	Tyr	Tyr-MB	Tyr	Tyr-MB	Tyr	Tyr-MB	Tyr	Tyr-MB
10	0.029 ± 0.002	0.162 ± 0.034	0.241 ± 0.069	1.451 ± 0.807	6.861 ± 0.456	18.973 ± 4.234	33.585 ± 13.185	13.400 ± 3.967
20	0.023 ± 0.001	0.146 ± 0.029	0.352 ± 0.063	3.794 ± 1.452	5.272 ± 0.281	17.157 ± 2.392	15.000 ± 0.802	4.525 ± 0.696
30	0.019 ± 0.001	0.176 ± 0.061	0.389 ± 0.061	8.842 ± 4.588	4.463 ± 0.236	21.360 ± 1.016	11.505 ± 0.227	2.403 ± 0.539

Kinetic parameters, the Michaelis–Menten constant (K_m_), and maximal velocity (V_max_) were obtained from GraphPad Prism software, version 8.1.2. The enzyme turnover (k_cat_) was defined as V_max_/E_t_, where E_t_ is the concentration of enzyme in mM. k_cat_/K_m_, enzyme efficiency. The measurements, which were completed in triplicates from 10 to 30 min at 10 min intervals, are shown.

## Data Availability

Data available in Supplementary Materials.

## Supplementary Materials

The supporting information can be downloaded at: https://www.mdpi.com/article/10.3390/ijms24010639/s1.

## Abbreviations

TYR: tyrosinase; TYRP2: tyrosinase-related protein 2; TYRP1: tyrosinase-related protein 1; OCA: oculocutaneous albinism; TYR-MB: TYR immobilized to magnetic beads; L-DOPA: levodopa; DHICA: 5,6-dihydroxyindole-2-carboxylic acid; IQCA: 5,6-indolequinone-2-carboxylic acid; DHI: 5,6-dihydroxyindole; IQ: indole-5,6-quinone; MBTH: 3-methyl-2-benzothiazolinone hydrazone hydrochloride; TCEP: tris(2-carboxyethyl)phosphine hydrochloride; TYR + L-DOPA: diphenol oxidation of L-DOPA; TYRP1 + DHICA: oxidation of DHICA to IQCA; TYRP2 + Dopachrome: dopachrome tautomerase reaction.
