# Supplementary material for: In Vitro Reconstitution of the Melanin Pathway’s Catalytic Activities Using Tyrosinase Nanoparticles
Source: Int J Mol Sci. 2022 Dec 30;24(1):639. doi: 10.3390/ijms24010639 (PMC9820814; doi:10.3390/ijms24010639)
Supplement: Supplementary file 1 [file ijms-24-00639-s001.zip › ijms-2088117-supplementary.pdf]

Isabella Osuna, Monika B. Dolinska, and Yuri V. Sergeev. *In vitro* reconstitution of catalytic activities of melanin pathway using tyrosinase nanoparticles.

Supplementary Material

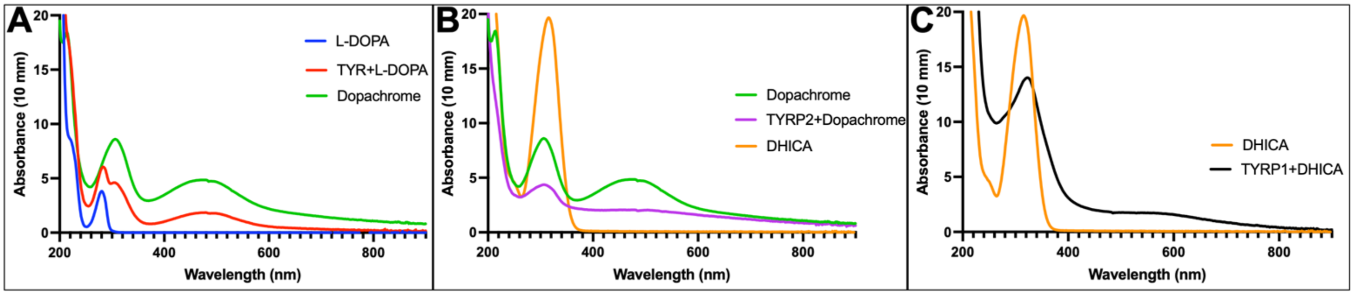

Supplementary Figure S1. TYR, TYRP2, and TYRP1 are enzymatically active. (A) The substrate and product involved in the first step catalyzed by TYR are spectrophotometrically shown. (B) Dopachrome tautomerase reaction, the substrate, and the product in the second step are shown spectrophotometrically. (C) The last step, oxidation of DHICA to IQCA by TYRP1, is shown spectrophotometrically.
